# Risk factors and preventive strategies for precocious puberty: a narrative review

**DOI:** 10.3389/fped.2026.1891119

**Published:** 2026-09-14

**Authors:** Jia-Qi Lin, Tian-Tian Huang, Ji-Cheng Zhang, Xiang-Yun Tang, Yue Chen, Xiao-Fan Wu, Xin Zhang, En-Ze Lei

**Affiliations:** 1 School of Laboratory Medicine, Hubei University of Chinese Medicine, Wuhan, China; 2 Department of Traditional Chinese Medicine Pediatrics, Maternal and Chid Health Hospital of Hubei Provincial, Women and Children’s Hospital of Hubei Provincial, Wuhan, China; 3 Department of Pediatrics, Zhongxiang Hospital of Traditional Chinese Medicine, Zhongxiang, China; 4 First Clinical College, Hubei University of Chinese Medicine, Wuhan, China; 5 Department of Pediatrics, Hubei Provincial Hospital of Traditional Chinese Medicine, Affiliated Hospital of Hubei University of Chinese Medicine, Wuhan, China; 6 Hubei Shizhen Laboratory, Wuhan, China; 7 Hubei Key Laboratory of Liver and Kidney Research and Application of Traditional Chinese Medicine, Wuhan, China

**Keywords:** children, narrative review, precocious puberty, prevention strategy, risk factor

## Abstract

Precocious puberty (PP) is a common pediatric endocrine disorder with rising global incidence, significantly compromising children's quality of life and long-term health. Current therapeutic options remain limited in applicability, leaving many patients inadequately controlled. Prevention targeting known risk factors is substantially more cost-effective than post-diagnosis treatment; however, the available evidence on PP risk factors remains fragmented. This review systematically synthesizes the risk factors for PP across seven age stages—preconception period, fetal period, infancy, toddlerhood, preschool age, school age, and adolescence—and eight domains: genetic predisposition and family history, maternal conditions and prenatal factors, delivery mode and early feeding patterns, dietary behaviors and nutritional status, individual lifestyle habits, family/school/community environments, early clinical signs and indicators, and pre-existing and idiopathic medical conditions. Corresponding preventive strategies, including optimizing parental reproductive health, promoting balanced nutrition, physical activity, and psychosocial well-being, and fostering multi-sector collaboration, are briefly discussed. This review aims to raise awareness of PP risk factors and facilitate the implementation of preventive strategies.

## 1 Introduction

Precocious puberty (PP) is a common endocrine disorder in children and is typically defined as the onset of secondary sexual characteristics before 7.5 years of age in girls and 9.0 years in boys (1). The adverse effects of PP during childhood are mainly manifested by the early appearance of secondary sexual characteristics (e.g., breast development and menarche in girls and testicular enlargement in boys) and associated psychological problems (e.g., depression and anxiety) (2). These adverse effects may persist into adulthood and are primarily manifested as reduced final adult height. In addition, PP may increase the risk of reproductive system cancers and cardiovascular diseases, posing a potential threat to long-term health (3, 4). During the COVID-19 pandemic, children and adolescents experienced prolonged home confinement, and their lifestyles changed markedly, including increased use of electronic devices, reduced physical activity space, disrupted sleep patterns, and extended time spent with parents or caregivers. Many studies worldwide have reported a significant increase in the incidence of PP, and a substantial rise in cases has been observed globally during this period (5, 6). Therefore, the prevention and management of PP have become important public health concerns (7).

The pathogenesis of PP is complex and influenced by multiple factors, including intracranial diseases, endocrine disorders, and environmental risk factors. In clinical practice, most cases of PP are idiopathic; however, recent studies suggest that genetic background plays an increasingly important role and represents a congenital factor that should not be overlooked (8). In addition, the incidence of PP increased more rapidly during the COVID-19 pandemic than before, suggesting that lifestyle factors are also important acquired determinants of the onset and progression of PP (9, 10) The primary treatment for central precocious puberty (CPP) currently involves gonadotropin-releasing hormone analogues, such as triptorelin and leuprolide (11). However, the clinical use of these agents is limited by strict indications and contraindications, a treatment duration that typically exceeds two years, and high costs, which restrict their widespread application (12).

Compared with clinical treatment after diagnosis, preventive interventions targeting potential risk factors for PP may provide greater economic and public health benefits (13). However, significant gaps remain in the prevention of PP. To date, studies on the risk factors for PP remain fragmented, and a clear and systematic framework has not yet been established, which hinders the development and implementation of effective prevention strategies (14). Therefore, this narrative review systematically summarizes the various influencing factors of PP and proposes that its risk factors should be considered across seven age groups and eight levels in Figure 1. Based on these risk factors, corresponding preventive measures are also briefly discussed to improve public awareness and promote the implementation of prevention strategies for PP. Ultimately, these efforts aim to support the physical and mental health of children and adolescents.

**Figure 1:**
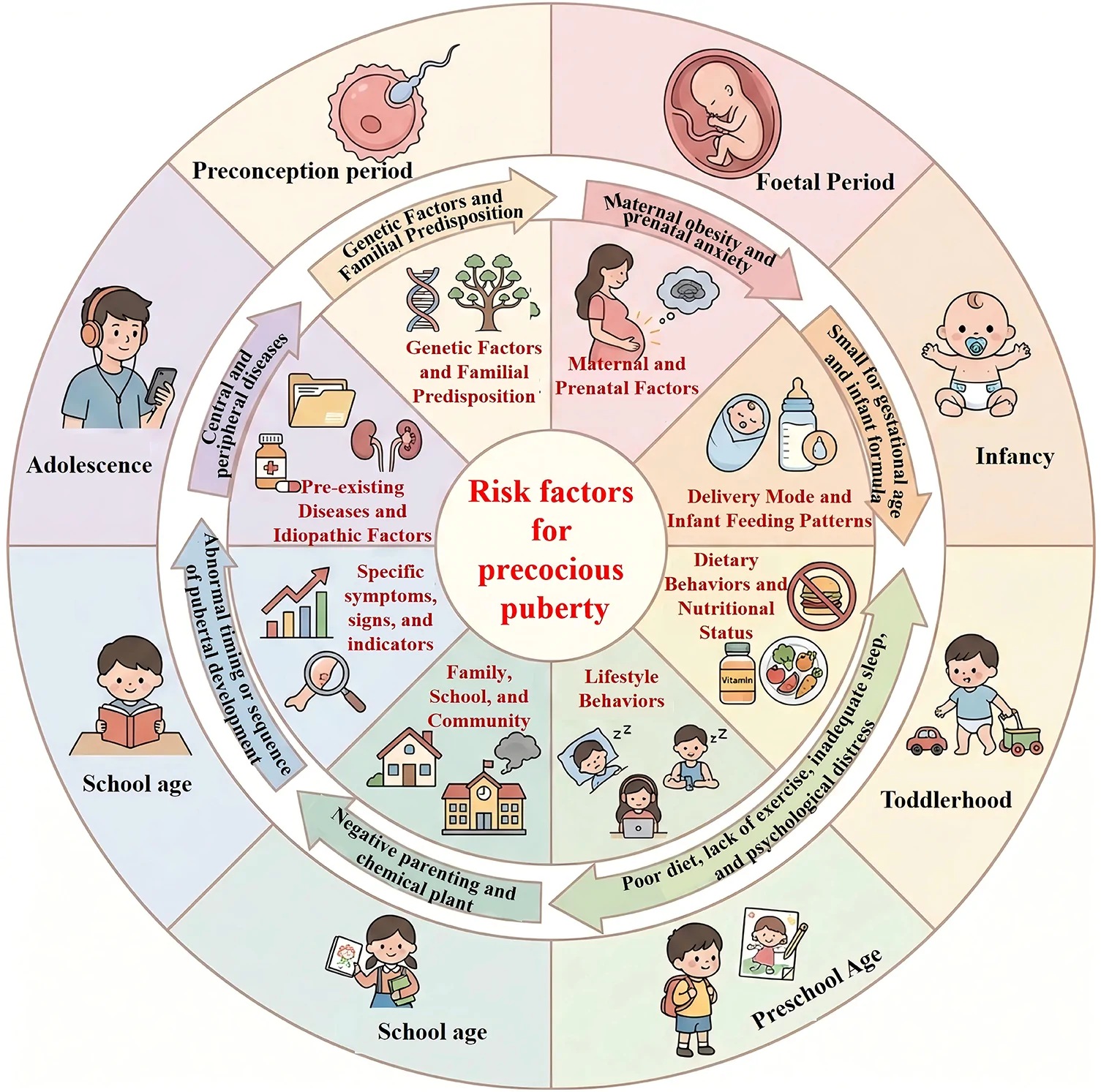
Hierarchy of risk factors for precocious puberty in children.

## 2 Genetic factors and familial predisposition

Genetic factors play a key role in endocrine diseases. CPP, caused by premature activation of the hypothalamic–pituitary–gonadal axis (HPGA), may result from pathogenic mutations in a single gene or the additive effects of multiple gene variants (15, 16). Similarly, family history, including familial CPP and neurodevelopmental disorders, may serve as potential clinical predictors of PP (17). According to the characteristics of genetic defects, the genetic factors associated with PP can be categorized into four groups: gene variants, epigenetic regulation, chromosomal variations, and gene regulatory networks. The most well-characterized single-gene functional mutations include gain-of-function mutations in the KISS1 and KISS1R genes and loss-of-function mutations in the MKRN3, DLK1, and LIN28 genes (15–18). Common gene variants, including non-functional or missense variants, have been identified in genes such as NAA10, PORCN, NR0B1, ABCD1, LHCGR, MECP2, and GATA4 (19–25). Large-scale genome-wide association studies (GWAS) have shown that single nucleotide polymorphisms in these genes are also closely associated with PP (26, 27). As shown in Table 1, studies on the genetic etiology of PP have shown that epigenetic mechanisms (such as DNA methylation and microRNA regulation), syndromes caused by chromosomal variations (structural or numerical), and the synergistic effects of multiple variant genes within gene regulatory networks also play important roles in the early onset of puberty (15, 28–38). Familial CPP is influenced by genetic factors and can be defined by early onset of sexual development or a clinical history of CPP in first-, second-, or third-degree relatives of affected individuals (39), which is an important factor for clinical consultation and screening of PP (Table 2). Familial CPP accounts for approximately 20–27.5% of CPP cases, with similar frequencies of maternal and paternal transmission (40).

**Table 1:** The heredity factors of PP.

Classification		Hereditary factors associated with PP	Refs.
Category	Subcategory		
Genetic variation	Functional gene mutation	Gain-of-function mutations (KISS1, KISS1R), Loss-of-function mutations (MKRN3, DLK1, LIN28), Gain-of-function mutations in the GNAS gene (McCune-Albright syndrome)	(15–18)
	Non-functional variant or missense variant	NAA10 (p.Arg83Cys), PORCN, NR0B1, ABCD1, LHCGR, MECP2 (Rett syndrome), GATA4	(19–25)
	Single nucleotide polymorphism (SNP)	KISS1 gene polymorphism (rs5780218、rs12998、rs10158616、rs10159082、rs7538038), SIRT1 gene polymorphism (rs7895833、rs3758391、rs3740051), VDR gene polymorphism (rs1544410、rs1544410、rs7975232 、rs731236), LIN28B gene polymorphism (rs7759938), ERα gene polymorphism(rs2234693)	(26–27)
Epigenetics	DNA methylation or demethylation	Methylation of the EED and CBX7 promoters, hypomethylation of the DLK1 gene, differentially methylated genes encoding zinc finger proteins, and loss of methylation at 11p15 ICR1 (Silver Russell syndrome)	(15, 28–29)
	MicroRNAs	Let-7、MiR-200/429、MiR-155、MiR-29 family、MiR-375、MiR-505-3p、MiR-199-3p、MiR-664-2、MiR-30b	(30)
Chromosomal variation	Structural or numerical variation	Temple syndrome(14q32.2 deletion)、Williams-Beuren syndrome (7q11.2 deletion)、Simpson–Golabi–Behmel syndrome (Xq26.2 deletion)、Prader-Willi syndrome (15.11q3 deletion)、Myhre syndrome (18q21.2 deletion)、Beckwith-Wiedemann syndrome (11p15.5 deletion)	(31–36)
Gene regulatory network	Regulatory network of chromatin, transcription factor, and gene interactions	G protein-coupled receptor network (KISS1R, TACR3, PROKR2, GNRHR, LHCGR, and FSHR), Combined regulation of TTF1 and EAP1	(37, 38)

**Table 2:** Risk factors of PP and related mechanism.

Classification		Certain risk factors of PP (Decrease ↓/Increase ↑)	Related mechanism	Refs.
Genetic Factors and Familial Predisposition	Genes and family history	Susceptibility genes ↑	Involving chromosomal inheritance, gene expression, and epigenetic regulation	(15–40)
		First-, second-, and third-degree relatives with PP ↑		
		Maternal menarche ≤10 years↑		
Maternal and Prenatal Factors	Maternal preconception health	Maternal overweight/obesity ↑	Maternal abnormal conditions, through intrauterine exposure and intergenerational transmission, induce precocious activation of the HPG axis or adrenarche	(41, 42)
		Maternal polycystic ovary syndrome ↑		
	Mode of conception	ART conception (in girls) ↑ Primiparity ↑	Infants conceived via ART have a higher risk of low birth weight than those conceived without ART, thereby indirectly increasing the risk of precocious puberty	(43)
	Prenatal living environment	Elevated EDC exposure (e.g., phthalates, parabens, BPA) ↑	Endocrine-disrupting chemicals interfere with estrogen and androgen signaling pathways, thereby accelerating pubertal development	(44–46)
	Maternal prenatal behavior	Prenatal healthy diet ↓ prenatal malnutrition ↑ prenatal circadian rhythm disruption ↑ prenatal anxiety ↑	Prenatal adverse behaviors exert long-term adverse effects on Kisspeptin-neurokinin B-dynorphin A neurons in the hypothalamic reproductive center of offspring	(47–50)
Delivery Mode and Infant Feeding Patterns	Delivery Mode	Cesarean section ↑ vaginal delivery ↓	Involving neonatal gut microbiota colonization	(51)
	Perinatal status	SGA ↑	During catch-up growth, SGA infants exhibit changes including increased visceral fat, decreased insulin sensitivity, and elevated IGF-1 concentrations	(52–55)
	Infant Feeding Patterns	Breastfeeding (for >6 months) ↓ formula feeding (especially soy-based formula) ↑	Breast milk indirectly shapes the early gut microbiota, whereas soy-based formula contains high concentrations of isoflavones that may exert potential estrogenic effects on infant development	(56, 57)
Dietary Behaviors and Nutritional Status	Dietary pattern	Traditional dietary pattern ↓	Unhealthy dietary behaviors or specific nutritional components induce adipose conversion and hormonal dysregulation	(58, 59)
		High-vegetable and high-protein dietary pattern ↓		
		High-animal-food and high-fruit dietary pattern ↑		
	Dietary habits	Poor overall dietary habits ↑		(60, 61)
		Binge eating ↑		
		High-frequency consumption of sugar-sweetened beverages ↑		
		Probiotic beverages ↓		
	Nutrients	Dietary fat ↑		(62–73)
		Polyunsaturated fatty acids		
		High carbohydrate ↑		
		Food additives (e.g., tartrazine, propolis, acesulfame K) ↑		
		Food additives (e.g., aspartame, glycyrrhizic acid, EGCG) ↓		
		Pesticide residues in food ↑		
Lifestyle Behaviors	Exercise habits	Prolonged use of electronic devices or sedentary behavior ↑	Through its effects on energy metabolism, hormone secretion, and related functions, a comprehensive influence is exerted on the timing of pubertal onset	(74–76)
		Aerobic training ↓		
	Sleep habits	Sleep disorder (narcolepsy type 1) ↑		(77–80)
		Longer sleep duration ↑		
		Insufficient sleep ↑		
	Psychological behavior	Depressive and anxiety status ↑Abnormal psychological behavior ↑		(81–86)
		ADHD ↑		
		ASD ↑		
Family, School, and Community Environments	Family environments	Family breakdown ↑	Common major environmental stressors can induce changes in childrens brain structure and physiological status	(87–92)
		Internationally adopted children ↑		
		Frequent interparental conflict ↑		
		Parental alcoholism ↑		
		Maternal anxiety and stress ↑		
		Childhood emotional abuse ↑		
		Child neglect ↑		
		Negative parenting styles ↑		
	School environments	Socioeconomic deprivation of the school area		(93)
	Community environments	Vulnerable community groups ↑		(14, 94–95)
		Residence near chemical industrial parks ↑		
Clinical Signs, Symptoms, and Indicators	Clinical Signs and Symptoms	Breast enlargement (in girls) ↑Vaginal bleeding (in girls) ↑ Testicular enlargement (in boys) ↑	Abnormal timing or sequence of pubertal development	(96–108)
		Hoarseness ↑		
		Growth spurt ↑		
		Overweight or obesity ↑		
		Fat mass index (in girls) ↑Increased systolic blood pressure (in boys) ↑		
		Childhood acne ↑		
	Indicators	Sudden increase in pituitary volume ↑		(109–115)
		Sudden increase in bone age ↑		
		Abnormal findings on breast, uterus, and adnexal ultrasonography ↑ abnormal serum biomarkers ↑		
Pre-existing Diseases and Idiopathic Diseases	Pre-existing Diseases	Antibiotic exposure ↑	Specific diseases directly or indirectly cause premature activation of the HPG axis	(116–118)
		Chronic kidney disease ↑Hidradenitis suppurativa ↑		
	Idiopathic Diseases	Thyroid dysfunction ↑ premature adrenarche ↑ congenital adrenal hyperplasia ↑ adrenocortical tumor ↑		(119–131)
		Leydig cell tumor ↑ granulosa cell tumor of the ovary ↑ ovarian cyst ↑ hypothalamic hamartoma ↑ pineal cyst ↑		
		Rathkes cleft cyst ↑ postoperative status of intracranial tumor ↑		

## 3 Maternal and prenatal factors

Hormonal imbalance in the intrauterine environment caused by abnormal maternal conditions or adverse prenatal factors may disrupt reproductive processes and directly affect the growth and development of offspring. Maternal conditions—including preconception health status, mode of conception, prenatal environment, and maternal behaviors—have been associated with the risk of PP in offspring (as shown in Table 2). Several epidemiological studies have shown that maternal overweight or obesity and polycystic ovary syndrome before pregnancy are two common factors associated with early menarche in daughters and earlier pubertal development in both sexes (41, 42). A cohort study based on Danish national data from 1985 to 2015 found that children conceived by assisted reproductive technology had a higher risk of developmental disorders during adolescence, with PP more commonly observed in girls and delayed puberty more frequently observed in boys (43). Environmental endocrine-disrupting chemicals, such as phthalates, are widely present in flexible plastic products, leading to widespread human exposure (44). Prenatal maternal exposure to phthalates and parabens may affect early pubertal development in boys, whereas prenatal exposure to bisphenol A may increase the risk of PP in girls (45, 46). Prenatal adverse maternal behaviors, such as malnutrition, circadian rhythm disruption, and anxiety, may have long-term effects on kisspeptin–neurokinin B–dynorphin neurons in the reproductive center of the hypothalamic arcuate nucleus in offspring, thereby indirectly contributing to the development of PP (47–49). In addition, several case–control studies have reported that certain factors are not significantly associated with PP or show inconsistent effects, including maternal hypertension, diabetes, maternal age, prenatal smoking, and alcohol consumption (50).

## 4 Delivery mode and infant feeding patterns

The mode of delivery may also influence the risk of PP in offspring. Cesarean section has been identified as a risk factor for PP, whereas vaginal delivery may have a protective effect (51). This may be because infants delivered by cesarean section are not exposed to maternal vaginal microbiota during birth, which may hinder the early establishment of a balanced gut microbiota. Several studies have shown that preterm birth is not significantly associated with early puberty (52). However, being born small for gestational age (SGA) or having low birth weight (<1 SD) has been identified as a risk factor for earlier pubertal onset. This association may be related to metabolic changes during catch-up growth in small-for-gestational-age infants, including increased visceral fat, decreased insulin sensitivity, and elevated IGF-1 concentrations (53–55). Differences between human milk and formula feeding have long been studied and may influence pubertal development (56). Existing studies, primarily focused on girls (as shown in Table 2), have found that formula-fed girls tend to experience earlier menarche than breastfed girls, whereas exclusive breastfeeding during the first 6 months of life appears to be an important protective factor against CPP. Girls fed soy-based formula, which contains high concentrations of isoflavones with potential estrogenic activity, may experience earlier breast development and earlier menarche (57).

## 5 Dietary behaviors and nutritional Status

Similar to infant feeding, the dietary behaviors and nutritional status of preschool- and school-aged children play important roles in determining the timing of pubertal onset (132). A cross-sectional study of Chinese primary school students aged 6–9 years found that, compared with Westernized and high-protein dietary patterns, the traditional dietary pattern was protective against PP (58). Another case–control study reported that dietary patterns rich in vegetables and protein were protective against PP, whereas dietary patterns high in animal foods and fruits were associated with an increased risk of PP in Chinese girls (59). Regarding dietary habits, unhealthy eating behaviors, such as binge eating and frequent consumption of sugar-sweetened beverages, may increase the risk of PP (60, 61). This may occur because such behaviors promote adiposity and hormonal imbalances, which can indirectly trigger premature activation of the HPGA. With socioeconomic development, overnutrition among children has become increasingly prevalent (62–64). Excessive intake of energy-dense nutrients, particularly fats and sugars, has been identified as a risk factor for PP (65, 66). Food safety issues may also contribute to the risk of PP (as shown in Table 2). On the one hand, certain food additives, such as tartrazine, propolis, and acesulfame potassium, have been reported as potential risk factors for PP (67–69), whereas others, including aspartate and glycyrrhizic acid, may exert protective effects (70–72). On the other hand, pesticide residues in food may act as endocrine-disrupting chemicals and contribute to the early onset of puberty in children (73).

## 6 Lifestyle behaviors

Similar to the mechanisms underlying obesity, prolonged use of electronic devices (>2 h/day) and a sedentary lifestyle may increase the risk of PP in school-aged children (74). Conversely, increased physical activity, regardless of exercise intensity, may help reduce the risk of PP, particularly in girls (73). A randomized controlled trial showed that 12 weeks of aerobic exercise had positive effects on improving pubertal development in children with CPP (75). Similarly, treatment with triptorelin combined with aerobic and resistance exercise demonstrated greater improvements in CPP than pharmacological therapy alone (76). Narcolepsy, a neurological disorder characterized by disturbed nocturnal sleep, is often comorbid with endocrine disorders, including obesity and CPP (77). An epidemiological survey conducted during the COVID-19 lockdown found that children with CPP had higher rates of sleep disorders and later bedtimes than healthy controls (78). Multiple cohort studies have shown that longer sleep duration is associated with a lower risk of premature puberty in children, whereas insufficient sleep increases the risk of PP, particularly in girls (79, 80). Notably, adverse psychological conditions, such as persistent depression and anxiety, or psychosocial stress, such as low self-esteem and low self-confidence, may contribute to PP and are often comorbid with childhood mental disorders, including attention-deficit/hyperactivity disorder and autism spectrum disorder (81–86).

## 7 Family, school, and community environments

Early puberty in children is considered to result from complex interactions between biological and environmental factors operating at the individual, family, school, and community levels (as shown in Table 2). Many clinicians and psychologists believe that childhood adversity may influence brain structure and physiological regulation in children, thereby contributing to the earlier onset of PP (87, 88). Separation from the family of origin (e.g., family breakdown or international adoption), adverse family environments (e.g., frequent parental conflict, family alcohol misuse, or maternal anxiety and stress), and negative parenting styles (e.g., emotional abuse, neglect, or harsh parenting) are common risk factors for PP (89–92). Currently, relatively few studies have examined the associations between school and community environments and PP, and most rely on questionnaire-based surveys. A survey based on the Korean Adolescent Risk Behavior Web-based Survey conducted between 2007 and 2015 showed that socioeconomic deprivation in school districts was associated with earlier pubertal development (93). A study examining the influence of community characteristics, race, and socioeconomic status on pubertal onset among females in the United States reported that disadvantaged community environments were associated with an increased risk of PP (94, 95). Another case–control study conducted in China suggested that living near a chemical industrial park was associated with an increased risk of PP (14).

## 8 Clinical signs, symptoms, and indicators

Normal puberty develops in stages, with relatively stable timing and sequence of characteristic developmental events, as shown in Figure 2. Clinically, breast enlargement or prepubertal vaginal bleeding may be the first signs of PP in girls (96, 97), whereas testicular enlargement is often the earliest sign in boys (98). Voice changes and rapid height growth are also noticeable indicators of pubertal development (99–101). Therefore, early recognition of these clinical manifestations, which may indicate the initiation of pubertal development, is critical for the early detection and prevention of PP. Several meta-analyses have demonstrated that long-term overweight and obesity during childhood are independent risk factors for PP, and weight control is an important strategy for its prevention (102, 103). This may occur because obesity-induced hypothalamic inflammation can promote the premature activation of gonadotropin-releasing hormone neurons, thereby contributing to the development of PP (104). However, the effects of fat mass index on PP differ between sexes, showing a positive association with sexual development in girls but a negative association in boys (105). Elevated systolic blood pressure and childhood acne have also been reported as risk factors for PP in boys (106–108). Consistent with current guidelines and consensus statements on PP (as shown in Table 2), increases in pituitary volume and bone age, breast enlargement detected by ultrasonography, uterine and adnexal enlargement on pelvic ultrasonography, and abnormalities in serum biomarkers—including sex hormones, vitamin D, IGF-1, IGFBP-3, inhibin B, dehydroepiandrosterone sulfate, and leptin—are important clinical indicators and diagnostic criteria for PP (109–115).

**Figure 2:**
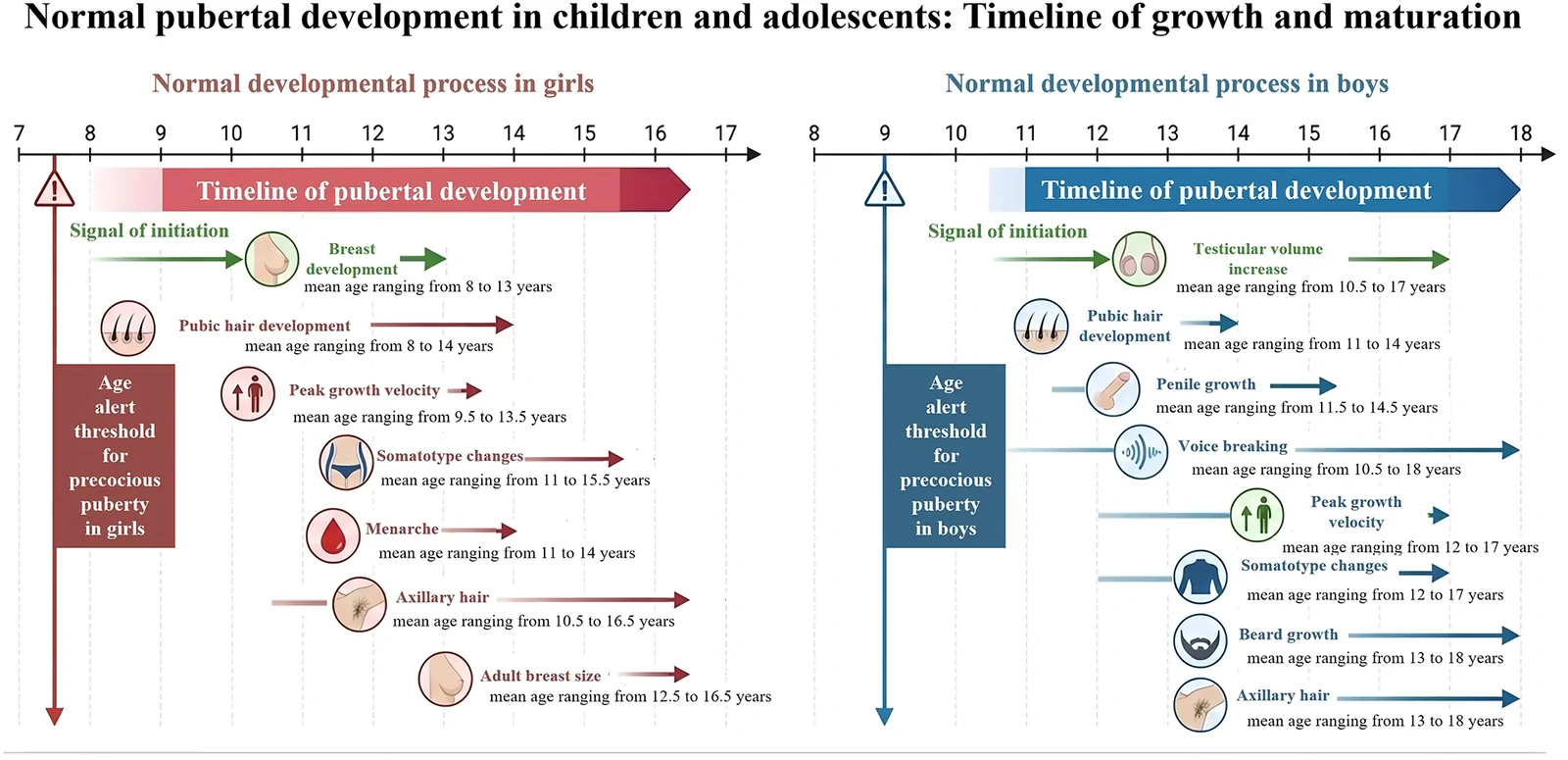
Diagram of normal pubertal development.

## 9 Pre-existing diseases and idiopathic factors

The HPGA is the core component of the endocrine regulatory network in children. It interacts with, and is regulated by, other neuroendocrine axes, including the hypothalamic–pituitary–adrenal axis, hypothalamic–pituitary–thyroid axis, and the growth hormone–insulin-like growth factor-1 axis. Through these interactions, the HPGA integrates nutritional, metabolic, and energy signals and ultimately regulates the initiation and progression of puberty. Consequently, disorders affecting these regulatory systems during childhood may contribute to the development of PP. Therefore, careful evaluation of pre-existing diseases and idiopathic conditions in children is important for the early screening and diagnosis of PP. Previous clinical reports have identified several medical conditions associated with an increased risk of PP, including antibiotic exposure (116), chronic kidney disease (117), hidradenitis suppurativa (118), thyroid dysfunction (119, 120), premature adrenarche (121), congenital adrenal hyperplasia (122), adrenocortical tumors (123), Leydig cell tumors of the testis (124), ovarian granulosa cell tumors (125), ovarian cysts (126), hypothalamic hamartoma (127), pineal cysts (128), Rathke cleft cysts (129), and a history of intracranial tumor surgery (130, 131).

## 10 Prevention strategies for PP based on risk factors

At present, the clinical understanding of PP and its prevention and management remains limited. Therefore, coordinated efforts among families, schools, healthcare systems, and communities are essential to reduce the occurrence of PP. With increasing knowledge of the pathogenesis and risk factors of PP, and considering the developmental characteristics of children at different ages, the following prevention strategies are proposed (some strategies still require further confirmation through evidence-based studies).

(1) Genetic screening and family history assessment

PP shows a genetic predisposition, with similar maternal and paternal inheritance patterns. A family history of early pubertal onset among third-degree relatives (e.g., early maternal menarche or early paternal spermarche), as well as syndromes caused by chromosomal abnormalities such as Williams–Beuren syndrome and Prader–Willi syndrome, may increase the risk of PP. Therefore, prenatal screening for genetic diseases and birth defects should be performed when appropriate and ethically permissible. In addition, obtaining a detailed family history of pubertal timing should be included as a routine component of clinical screening.

(2) Maternal health and prenatal care

Maternal health before and during pregnancy plays a critical role in the growth and development of offspring. Particular attention should be paid to maternal overweight/obesity and polycystic ovary syndrome, both of which may affect fetal endocrine programming. Appropriate medical interventions should be undertaken to restore normal reproductive and metabolic conditions before conception. During pregnancy, adopting a balanced diet, regular sleep–wake patterns, avoidance of environmental endocrine disruptors, and effective management of prenatal stress and anxiety may support placental function and promote healthy fetal development.

(3) Perinatal factors and early-life feeding

Natural conception without assisted reproductive technologies, vaginal delivery, and breastfeeding may help reduce the risk of PP. A balanced vaginal microbiota supports early neuroendocrine development, while breast milk provides essential nutrients and may delay exposure to exogenous estrogenic substances. Infants born SGA or with low birth weight should undergo long-term monitoring of growth and development until puberty. Particular attention should be paid to catch-up growth, during which Tanner staging of sexual development should be regularly assessed.

(4) Healthy lifestyle during childhood

The preschool and school-age periods are critical stages for establishing dietary, physical activity, sleep, and psychological habits. Maintaining balanced nutrition, healthy eating behaviors, regular sleep patterns, moderate physical activity, and psychological well-being are essential for preventing PP. From a nutritional perspective, it is important to prevent overnutrition, excessive caloric intake, and consumption of foods with high pesticide residues. In terms of physical activity, children should avoid prolonged sedentary behavior, particularly excessive use of electronic devices, and engage in regular aerobic exercise. Following established pediatric sleep hygiene guidelines can help ensure sufficient sleep duration and quality. Psychologically, children should be encouraged to maintain a positive emotional state, and professional medical support should be sought if mental health problems arise.

(5) Active health management and community involvement

The concept of active health, which emphasizes the transition from passive disease treatment to proactive prevention and health management, represents a new paradigm in modern medicine. Families, schools, communities, and healthcare institutions play key roles in children's active health management. The family environment is particularly important. Harmonious family relationships and positive parenting styles can reduce psychosocial stress and support healthy development. Parents also play a crucial role in monitoring children's growth and pubertal development. Meanwhile, schools and communities should provide safe, healthy, and environmentally friendly environments. Public health education activities—such as lectures and informational materials—can improve awareness of PP and promote active health management (as shown in Figure 1).

(6) Early detection and comprehensive prevention

The prevention of PP requires a multidisciplinary and systematic approach. Improving public awareness of the normal timing and sequence of pubertal development (as shown in Figure 2) can help parents, teachers, and community healthcare providers recognize early signs of abnormal development. Given the limited time window for pubertal development and the complex etiology of PP, early detection, early intervention, and early treatment remain the most effective strategies for prevention and management.

## 11 Summary

This review systematically summarizes the risk factors associated with PP in children. Multiple factors contribute to the susceptibility to PP, including genetic predisposition and family history, maternal conditions and prenatal factors, delivery mode and early feeding patterns, dietary behaviors and nutritional status, individual lifestyle habits, family, school and community environments, early clinical signs and indicators, as well as pre-existing and idiopathic medical conditions. Although certain determinants—such as genetic background, chromosomal variations, and fetal sex—cannot currently be modified, parents can reduce the risk of PP by maintaining a healthy reproductive status and lifestyle before and during pregnancy. In addition, establishing balanced nutritional intake, regular physical activity, and adequate sleep during childhood may help prevent early pubertal onset while also reducing the risk of other chronic diseases. Furthermore, promoting psychological well-being in children and improving their living and learning environments are important societal measures for the prevention of PP. Collaborative efforts from families, schools, healthcare systems, and communities are essential to reduce the incidence of PP and alleviate the long-term health and economic burden associated with endocrine disorders. Future large-scale prospective studies are needed to further clarify the causal relationships between environmental, metabolic, and genetic factors and the onset of precocious puberty, which may provide stronger evidence for targeted prevention strategies.

## References

[1] Alghamdi A. Precocious puberty: types, pathogenesis and updated management. Cureus. (2023) 15(10):e47485. 10.7759/cureus.47485 38021712 PMC10663169

[2] Lee PA Fuqua JS. Abnormal puberty revisited: a practical approach. Endocrinol Metab Clin North Am. (2024) 53(2):xi–xii. 10.1016/j.ecl.2024.03.002 38677874

[3] Nguyen AH Hurwitz M Sullivan SA Saad A Kennedy JLW Sharma G. Update on sex specific risk factors in cardiovascular disease. Front Cardiovasc Med. (2024) 11:1352675. 10.3389/fcvm.2024.1352675 38380176 PMC10876862

[4] Cheng TS Ong KK Biro FM. Adverse effects of early puberty timing in girls and potential solutions. J Pediatr Adolesc Gynecol. (2022) 35(5):532–5. 10.1016/j.jpag.2022.05.005 35644513

[5] Zhang J Xu J Tang X Wu R. Comparison of central precocious puberty frequency before and during COVID-19: a systematic review and meta-analysis. BMC Endocr Disord. (2024) 24(1):219. 10.1186/s12902-024-01749-4 39420314 PMC11484383

[6] Hoskyns RB Howard SR. Effects of the COVID-19 pandemic on the incidence of central precocious puberty; a narrative review. J Pediatr Endocrinol Metab. (2024) 37(2):102–9. 10.1515/jpem-2023-0507 38097507

[7] Kilberg MJ Vogiatzi MG. Approach to the patient: central precocious puberty. J Clin Endocrinol Metab. (2023) 108(8):2115–23. 10.1210/clinem/dgad081 36916130

[8] Zevin EL Eugster EA. Central precocious puberty: a review of diagnosis, treatment, and outcomes. Lancet Child Adolesc Health. (2023) 7(12):886–96. 10.1016/S2352-4642(23)00237-7 37973253

[9] Nguyen NN Do TD Truong HH Mai AN Chen YC. Difference in precocious puberty between pre-COVID-19 and COVID-19 periods: a meta-analysis. Am J Epidemiol. (2025) 194(4):1131–9. 10.1093/aje/kwae295 39168833

[10] Alonso GF. Precocious puberty, pandemic and beyond. Pituitary. (2024) 27(6):916–24. 10.1007/s11102-024-01434-8 39060817

[11] Kim SJ Kim JH Hong YH Chung IH Lee EB Kang E. 2022 Clinical practice guidelines for central precocious puberty of Korean children and adolescents. Ann Pediatr Endocrinol Metab. (2023) 28(3):168–77. 10.6065/apem.2346168.084 37798893 PMC10556443

[12] Breidbart E Ilkowitz J Regelmann MO Ashraf AP Gourgari E Kamboj MK. Precocious puberty and GnRH analogs: current treatment practices and perspectives among US pediatric endocrinologists. Horm Res Paediatr. (2025) 98(5):491–502. 10.1159/000539011 38718766 PMC12416872

[13] Lorenzon B Burlo F Barbi L Tamaro G Tornese G. Fifteen-minute consultation: the approach to the child with precocious puberty. Arch Dis Child Educ Pract Ed. (2024) 109(6):271–5. 10.1136/archdischild-2023-326217 38514136

[14] Dong Y Dai L Dong Y Wang N Zhang J Liu C. Analysis of risk factors of precocious puberty in children. BMC Pediatr. (2023) 23(1):456. 10.1186/s12887-023-04265-x 37697362 PMC10494349

[15] d&Amp;Apos;Aniello F Mariniello K Al Sayed Y Bhavsar K Read JE Guasti L. The role of DLK1 deficiency in central precocious puberty and association with metabolic dysregulation. Horm Res Paediatr. (2026) 99(1):38–48. 10.1159/000541554 PMC12795536 39419009

[16] Tajima T. Genetic causes of central precocious puberty. Clin Pediatr Endocrinol. (2022) 31(3):101–9. 10.1297/cpe.2022-0021 35928377 PMC9297165

[17] Canton APM Seraphim CE Montenegro LR Krepischi ACV Mendonca BB Latronico AC. The genetic etiology is a relevant cause of central precocious puberty. Eur J Endocrinol. (2024) 190(6):479–88. 10.1093/ejendo/lvae063 38857188

[18] Ertl D-A Ratzinger-Stoeger G Raimann A Anzengruber M Skoll K Gabor F. Successful use of metyrapone suppositories in an infant with neonatal cushing and McCune albright syndrome: a case report. Horm Res Paediatr. (2023) 98(1):1–6. 10.1159/000535266 38008082 PMC11854969

[19] Wojciechowska K Zie W Pietrzyk A Lejman M. A four-year-old girl with pathogenic variant in the NAA10 gene and precocious puberty - case report and literature review. Ann Agric Environ Med. (2024) 31(2):306–10. 10.26444/aaem/171758 38940118

[20] Akalın A Grzeschik KH Utine E Boduroğlu K Şimşek-Kiper PÖ. A long-term follow-up of a patient with a novel PORCN variant and additional clinical features. Mol Syndromol. (2024) 15(3):217–24. 10.1159/000535681 PMC11149962 38841326

[21] Gupta P Sharma R Jain V. Adrenal hypoplasia congenita-hypogonadotropic hypogonadism syndrome due to NR0B1 gene mutations. Indian J Pediatr. (2022) 89(6):587–90. 10.1007/s12098-021-04055-2 35230670

[22] Zhao C Zhu H Wang J Liu W Xue Y Hu Y. Central precocious puberty in a boy with X-linked adrenoleukodystrophy caused by a novel ABCD1 mutation. Heliyon. (2024) 10(7):e28987. 10.1016/j.heliyon.2024.e28987 38596053 PMC11002235

[23] Ha J Choi Y Jung MK Yoo EG Yoo HW. Familial male-limited precocious puberty due to an activating mutation of the LHCGR: a case report and literature review. Ann Pediatr Endocrinol Metab. (2024) 29(1):60–6. 10.6065/apem.2346042.021 38461807 PMC10925783

[24] Yang L Jiang M Yu R Hu R Xiong F Li J. A case report of precocious puberty related to rett syndrome and a literature review. Pharmazie. (2021) 76(11):559–61. 10.1691/ph.2021.1747 34782041

[25] Aversa T Luppino G Corica D Pepe G Valenzise M Coco R. A rare case of precocious puberty in a child with a novel GATA-4 gene mutation: implications for disorders of sex development (DSD) and review of the literature. Genes. (2023) 14(8):1631. 10.3390/genes14081631 37628683 PMC10454567

[26] Li Y Zhang H Li Q Huang X Kong X. Association of the KISS1, LIN28B, VDR and ER*α* gene polymorphisms with early and fast puberty in Chinese girls. Gynecol Endocrinol. (2023) 39(1):2181653. 10.1080/09513590.2023.2181653 36828304

[27] Dong L Zhou W Lin Z Tang L Deng X Chen B. Polymorphism rs5780218, rs12998 and rs10158616 in KISS1 gene among the Hubei province Chinese girls with the central precocious puberty. Endocrine. (2024) 84(3):1229–37. 10.1007/s12020-024-03716-4 38358556

[28] Palumbo S Palumbo D Cirillo G Giurato G Aiello F Miraglia del Giudice E. Methylome analysis in girls with idiopathic central precocious puberty. Clin Epigenetics. (2024) 16(1):82. 10.1186/s13148-024-01683-1 38909248 PMC11193236

[29] Ponce D Rodríguez F Miranda JP Binder AM Santos JL Michels KB. Differential methylation pattern in pubertal girls associated with biochemical premature adrenarche. Epigenetics. (2023) 18(1):2200366. 10.1080/15592294.2023.2200366 37053179 PMC10114989

[30] Jeong HR Hwang IT. The role of MicroRNAs as fine-tuners in the onset of puberty: a comprehensive review. Ann Pediatr Endocrinol Metab. (2024) 29(4):211–9. 10.6065/apem.2346238.119 39231482 PMC11374517

[31] Juriaans AF Kerkhof GF Mahabier EF Sas TCJ Zwaveling-Soonawala N Touwslager RNH. Temple syndrome: clinical findings, body composition and cognition in 15 patients. J Clin Med. (2022) 11(21):6289. 10.3390/jcm11216289 36362517 PMC9656486

[32] De Oliveira KMK Simões LO Dos Santos AM Steiner CE. Clinical findings in a series of thirty eight patients with williams-beuren syndrome. Cytogenet Genome Res. (2024) 164(3-4):1–9. 10.1159/000540941 39159616

[33] Watanabe K Noguchi A Takahashi I Yamada M Suzuki H Takenouchi T. Precocious puberty in a case of simpson-golabi-behmel syndrome with a *de novo* 240-kb deletion including GPC3. Hum Genome Var. (2022) 9(1):23. 10.1038/s41439-022-00196-8 35680838 PMC9184466

[34] Passone CGB Aragão LFF Franco RR Leite JES Gonzalez MAB Schil PSA. Puberty in girls with prader-willi syndrome: cohort evaluation and clinical recommendations in a Latin American tertiary center. Front Endocrinol. (2024) 15:1403470. 10.3389/fendo.2024.1403470 PMC11222560 38966214

[35] Lim D Kim JH Lee J. Myhre syndrome: the first case in Korea. Ann Pediatr Endocrinol Metab. (2021) 26(3):210–4. 10.6065/apem.2040214.107 34015905 PMC8505036

[36] Ye M Reyes Palomares A Iwarsson E Oberg AS Rodriguez-Wallberg KA. Imprinting disorders in children conceived with assisted reproductive technology in Sweden. Fertil Steril. (2024) 122(4):706–14. 10.1016/j.fertnstert.2024.05.168 38825304

[37] Suzuki E Miyado M Kuroki Y Fukami M. Genetic variants of G-protein coupled receptors associated with pubertal disorders. Reprod Med Biol. (2023) 22(1):e12515. 10.1002/rmb2.12515 37122876 PMC10134480

[38] Cukier P Wright H Rulfs T Silveira LFG Teles MG Mendonca BB. Molecular and gene network analysis of thyroid transcription factor 1 (TTF1) and enhanced at puberty (EAP1) genes in patients with GnRH-dependent pubertal disorders. Horm Res Paediatr. (2013) 80(4):257–66. 10.1159/000354643 24051510

[39] Tinano FR Canton APM Montenegro LR De Castro Leal A Faria AG Seraphim CE. Clinical and genetic characterization of familial central precocious puberty. J Clin Endocrinol Metab. (2023) 108(7):1758–67. 10.1210/clinem/dgac763 36611250

[40] Harbulot C Lessim S Simon D Martinerie L Storey C Ecosse E. Prevalence and clinical characteristics of isolated forms of central precocious puberty: a cohort study at a single academic center. Eur J Endocrinol. (2021) 184(2):243–51. 10.1530/EJE-20-0862 33156813

[41] Cinzori ME Strakovsky RS. Effects of elevated maternal adiposity on offspring reproductive health: a perspective from epidemiologic studies. J Endocr Soc. (2022) 7(1):bvac163. 10.1210/jendso/bvac163 36438545 PMC9683496

[42] Lunddorf LLH Arendt LH Ernst A Brix N Knudsen UB Olsen J. Maternal polycystic ovarian syndrome and pubertal development in daughters and sons: a population-based cohort study. Hum Reprod. (2022) 37(11):2623–34. 10.1093/humrep/deac197 36099165

[43] Klemetti R Perry B Henningsen AKA Spangmose AL Pinborg A Opdahl S. Puberty disorders among ART-conceived singletons: a nordic register study from the CoNARTaS group. Hum Reprod. (2022) 37(10):2402–11. 10.1093/humrep/deac192 36029044 PMC9527471

[44] Ma Y Mu X Gao R Zhang Y Geng Y Chen X. Maternal exposure to dibutyl phthalate regulates MSH6 crotonylation to impair homologous recombination in fetal oocytes. J Hazard Mater. (2023) 455:131540. 10.1016/j.jhazmat.2023.131540 37167869

[45] Curi TZ Passoni MT Lima Tolouei SE De Araújo Ramos AT França de Almeira SC Scinskas ABAF. Reproductive toxicity following in utero and lactational exposure to a human-relevant phthalate mixture in rats. Toxicol Sci. (2024) 197(1):1–15. 10.1093/toxsci/kfad102 37788136

[46] Freire C Castiello F Babarro I Anguita-Ruiz A Casas M Vrijheid M. Association of prenatal exposure to phthalates and synthetic phenols with pubertal development in three European cohorts. Int J Hyg Environ Health. (2024) 261:114418. 10.1016/j.ijheh.2024.114418 38968838

[47] Minabe S Iwata K Watanabe Y Ishii H Ozawa H. Long-term effects of prenatal undernutrition on female rat hypothalamic KNDy neurons. Endocr Connect. (2023) 12(1):e220307. 10.1530/EC-22-0307 36408965 PMC9782422

[48] Guan Y Xu M Zhang Z Liu C Zhou J Lin F. Maternal circadian disruption before pregnancy impairs the ovarian function of female offspring in mice. Sci Total Environ. (2023) 864:161161. 10.1016/j.scitotenv.2022.161161 36572306

[49] Fox MM Hahn-Holbrook J Sandman CA Marino JA Glynn LM Davis EP. Mothers’ prenatal distress accelerates adrenal pubertal development in daughters. Psychoneuroendocrinology. (2024) 160:106671. 10.1016/j.psyneuen.2023.106671 38000239 PMC11802187

[50] Dalmartello M Chiaffarino F Esposito G Cipriani S Ricci E La Vecchia C. A systematic review on maternal and perinatal factors influencing breast development. Early Hum Dev. (2023) 183:105816. 10.1016/j.earlhumdev.2023.105816 37421688

[51] Bigambo FM Wang D Niu Q Zhang M Mzava SM Wang Y. The effect of environmental factors on precocious puberty in children: a case-control study. BMC Pediatr. (2023) 23(1):207. 10.1186/s12887-023-04013-1 37127587 PMC10149633

[52] Suikkanen J Nurhonen M Cole TJ Paalanne M Matinolli H-M Tikanmäki M. Preterm birth and subsequent timing of pubertal growth, menarche, and voice break. Pediatr Res. (2022) 92(1):199–205. 10.1038/s41390-021-01690-5 34429512 PMC9411060

[53] Sabu S Corman H Noonan K Reichman NE Kuhn KB Radovick S. Small for gestational age and age at menarche in a contemporary population-based U.S. Sample. PLoS One. (2024) 19(9):e0309363. 10.1371/journal.pone.0309363 39240976 PMC11379201

[54] Peng Q Qiu W Li Z Zhao J Zhu C. Fetal genetically determined birth weight plays a causal role in earlier puberty timing: evidence from human genetic studies. Hum Reprod. (2024) 39(4):792–800. 10.1093/humrep/deae019 38384258

[55] Aghaee S Quesenberry CP Deardorff J Kushi LH Greenspan LC Ferrara A. Associations between infant growth and pubertal onset timing in a multiethnic prospective cohort of girls. BMC Pediatr. (2022) 22(1):171. 10.1186/s12887-022-03242-0 35361165 PMC8969386

[56] Kale A Deardorff J Lahiff M Laurent C Greenspan LC Hiatt RA. Breastfeeding versus formula-feeding and girls’ pubertal development. Matern Child Health J. (2015) 19(3):519–27. 10.1007/s10995-014-1533-9 24916206 PMC4263683

[57] Calcaterra V Cena H Sottotetti F Rossi V Loperfido F Zuccotti G. Breast and formula milk and early puberty onset. Children. (2023) 10(10):1686. 10.3390/children10101686 37892349 PMC10605833

[58] Chen X Fu S Chen C Yuan Y Dai Z Chen A. Association of traditional dietary pattern with early and precocious puberty: a population-based cross-sectional study. Pediatr Res. (2024) 96(1):245–52. 10.1038/s41390-024-03110-w 38431666

[59] Gu Q Wu Y Feng Z Chai Y Hou S Yu Z. Dietary pattern and precocious puberty risk in Chinese girls: a case-control study. Nutr J. (2024) 23(1):14. 10.1186/s12937-024-00916-6 38291391 PMC10829199

[60] Du Y Yan W Bigambo FM Zhou Q Ma C Gu W. Association between dietary behavior and puberty in girls. BMC Pediatr. (2024) 24(1):349. 10.1186/s12887-024-04840-w 38773477 PMC11106965

[61] Tsai MC Lee YL Chen YC. Association of the consumption of common drinks with early puberty in both sexes. Front Public Health. (2022) 10:854477. 10.3389/fpubh.2022.854477 36536777 PMC9758723

[62] Calcaterra V Verduci E Magenes VC Pascuzzi MC Rossi V Sangiorgio A. The role of pediatric nutrition as a modifiable risk factor for precocious puberty. Life. (2021) 11(12):1353. 10.3390/life11121353 34947884 PMC8706413

[63] Liang X Huang K Dong G Chen R Chen S Zheng R. Current pubertal development in Chinese children and the impact of overnutrition, lifestyle, and perinatal factors. J Clin Endocrinol Metab. (2023) 108(9):2282–9. 10.1210/clinem/dgad102 36881937

[64] Huang X-Y Chen J-X Ren Y Luo H-L Xiang W He X-J. Postnatal feeding with high-fat combined with high-glucose diet induces precocious puberty in sprague‒dawley rat pups. Biochem Biophys Res Commun. (2024) 693:149199. 10.1016/j.bbrc.2023.149199 38118311

[65] Tang J Xue P Huang X Lin C Liu S. Diet and nutrients intakes during infancy and childhood in relation to early puberty: a systematic review and meta-analysis. Nutrients. (2022) 14(23):5004. 10.3390/nu14235004 36501034 PMC9739867

[66] Xu Y Xiong J Gao W Wang X Shan S Zhao L. Dietary fat and polyunsaturated fatty acid intakes during childhood are prospectively associated with puberty timing independent of dietary protein. Nutrients. (2022) 14(2):275. 10.3390/nu14020275 35057456 PMC8778261

[67] Mindang ELN Awounfack CF Ndinteh DT Krause RWM Njamen D. Effects of tartrazine on some sexual maturation parameters in immature female wistar rats. Int J Environ Res Public Health. (2022) 19(16):10410. 10.3390/ijerph191610410 36012044 PMC9408620

[68] Polat R Çokluk E Budak Ö Tuncer FB. Effect of propolis on precocious puberty in female rats. J Clin Res Pediatr Endocrinol. (2022) 14(4):415–21. 10.4274/jcrpe.galenos.2022.2022-1-18 35770944 PMC9724062

[69] Wu HT Chiang CC Wang CT Chen YH Hsu SY Chen YC. Consumption of the nonnutritive sweetener acesulfame potassium increases central precocious puberty risk. J Hazard Mater. (2024) 461:132529. 10.1016/j.jhazmat.2023.13252 37738850

[70] Lin CY Nguyen NN Tsai WL Hsieh RH Wu HT Chen YC. Aspartame intake delayed puberty onset in female offspring rats and girls. Mol Nutr Food Res. (2024) 68(5):e2300270. 10.1002/mnfr.202300270 38389198

[71] Nguyen NN Lin C-Y Tsai W-L Huang H-Y Chen C-M Tung Y-T. Natural sweetener glycyrrhizin protects against precocious puberty by modulating the gut microbiome. Life Sci. (2024) 350:122789. 10.1016/j.lfs.2024.122789 38848942

[72] Gu Q Wang X Xie L Yao X Qian L Yu Z. Green tea catechin EGCG could prevent obesity-related precocious puberty through NKB/NK3R signaling pathway. J Nutr Biochem. (2022) 108:109085. 10.1016/j.jnutbio.2022.109085 35691596

[73] Sakali AK Bargiota A Fatouros IG Jamurtas A Macut D Mastorakos G. Effects on puberty of nutrition-mediated endocrine disruptors employed in agriculture. Nutrients. (2021) 13(11):4184. 10.3390/nu13114184 34836437 PMC8622967

[74] Chioma L Bizzarri C Verzani M Fava D Salerno M Capalbo D. Sedentary lifestyle and precocious puberty in girls during the COVID-19 pandemic: an Italian experience. Endocr Connect. (2022) 11(2):e210650. 10.1530/EC-21-0650 35029543 PMC8859940

[75] Cheng TS Brage S Van Sluijs EMF Ong KK. Pre-pubertal accelerometer-assessed physical activity and timing of puberty in British boys and girls: the millennium cohort study. Int J Epidemiol. (2023) 52(5):1316–27. 10.1093/ije/dyad063 37208864 PMC10555885

[76] Shokri E Heidarianpour A Razavi Z. Positive effect of combined exercise on adipokines levels and pubertal signs in overweight and obese girls with central precocious puberty. Lipids Health Dis. (2021) 20(1):152. 10.1186/s12944-021-01588-5 34742317 PMC8571828

[77] Casale S Assirelli V Pizza F Balsamo A Gennari M Pession A. Auxological and endocrine findings in narcolepsy type 1: seventeen-year follow-up from a pediatric endocrinology center. Front Endocrinol. (2023) 14:1037398. 10.3389/fendo.2023.1037398 PMC10311432 37396177

[78] Umano GR Maddaluno I Riccio S Lanzaro F Antignani R Giuliano M. Central precocious puberty during COVID-19 pandemic and sleep disturbance: an exploratory study. Ital J Pediatr. (2022) 48(1):60. 10.1186/s13052-022-01256-z 35461296 PMC9034068

[79] Gunawan SP Huang S-Y Wang C-C Huynh LBP Nguyen NN Hsu S-Y. Sleep deprivation alters pubertal timing in humans and rats: the role of the gut microbiome. Sleep. (2024) 47(2):zsad308. 10.1093/sleep/zsad308 38065690

[80] Mozafarian N Yazdi M Hashemipour M Hovsepian S Maracy MR. Association between sleep duration and early pubertal timing in children and adolescents: a systematic review and meta-analysis. Curr Pediatr Rev. (2023) 19(3):318–28. 10.2174/1573396318666220819145346 35986543

[81] Glass DJ Geerkens JT Martin MA. Psychosocial and energetic factors on human female pubertal timing: a systematized review. Evol Hum Sci. (2022) 4:e28. 10.1017/ehs.2022.24 37588922 PMC10426011

[82] Lee J Lee SI Lee YM Hong YH. Prevalence of attention deficit hyperactivity disorder in girls with central precocious puberty. J Atten Disord. (2023) 27(13):1460–6. 10.1177/10870547231180116 37449382

[83] Moriuchi Y Fuchigami T Horie M Yamada R Morioka I. Central precocious puberty (CPP) in two girls with autism Spectrum disorder (ASD). Cureus. (2023) 15(3):e35671. 10.7759/cureus.35671 37012946 PMC10066061

[84] Kardaş B Kardaş Ö Demiral M Özbek MN. Early puberty paradox: an investigation of anxiety levels of mothers and children, children’s quality of life, and psychiatric diagnoses. Eur J Pediatr. (2023) 182(8):3775–83. 10.1007/s00431-023-05057-2 37335399

[85] Lian Q Li R Elgar FJ Su Q. Early physical maturation and subjective health complaints in adolescent girls: a pooled cross-sectional analysis. J Epidemiol Community Health. (2023) 77(2):108–14. 10.1136/jech-2022-219547 36450457

[86] Xie LL Yang Y Xiong XY Yang L Wu X Zhang DG. A clinical study of girls with idiopathic central precocious puberty and psychological behavior problems. Clin Pediatr. (2023) 62(8):914–8. 10.1177/00099228221149551 36661073

[87] Ellis BJ Figueredo AJ Brumbach BH Schlomer GL. Fundamental dimensions of environmental risk: the impact of harsh versus unpredictable environments on the evolution and development of life history strategies. Hum Nat. (2009) 20(2):204–68. 10.1007/s12110-009-9063-7 25526958

[88] Pai LF Wang DS Hsu WF Huang SW Chung CH Chen SJ. New insights into precocious puberty and ADHD: a nationwide cohort study. Pediatr Res. (2024) 95(4):1157. 10.1038/s41390-023-02692-1 37316709

[89] Kwon A Cho YI Kim HJ Suh J Kim DH. The mediating effects of parenting style on the relationship between parental stress and behavioral problems in girls with precocious puberty in Korea: a cross-sectional study. BMC Pediatr. (2023) 23(1):352. 10.1186/s12887-023-04172-1 37438739 PMC10337196

[90] Rahimi F Mirghafourvand M Farvareshi M Yavarikia P. The effect of cognitive behavioral therapy on stress and anxiety of mothers of girls with precocious puberty symptoms: a randomized controlled trial. BMC Psychiatry. (2023) 23(1):738. 10.1186/s12888-023-05216-7 37817169 PMC10565989

[91] Steger V Stadelmann S White L Döhnert M. Child abuse and pubertal timing: what is the role of child sex and identity of the perpetrator? BMC Psychiatry. (2024) 24(1):242. 10.1186/s12888-024-05683-6 38561781 PMC10983734

[92] Young Ahn H Hee Yoo K Ryeong Song M. Guilt, uncertainty, education needs, and knowledge of mothers with children experiencing precocious puberty. Glob Pediatr Health. (2022) 9:2333794X221098305. 10.1177/2333794X221098305 35586592 PMC9109481

[93] Kim T Yun JW Son M Kim CB Choe SA. Age at menarche of adolescent girls and the neighbourhood socioeconomic status of their school area. Eur J Contracept Reprod Health Care. (2023) 28(1):65–71. 10.1080/13625187.2022.2104834 36053277

[94] Acker J Mujahid M Aghaee S Gomez S Shariff-Marco S Chu B. Neighborhood racial and economic privilege and timing of pubertal onset in girls. J Adolesc Health. (2023) 72(3):419–27. 10.1016/j.jadohealth.2022.10.013 36528517 PMC10505041

[95] Senger-Carpenter T Seng J Herrenkohl TI Marriott D Chen B Voepel-Lewis T. Applying life history theory to understand earlier onset of puberty: an adolescent brain cognitive development cohort analysis. J Adolesc Health. (2024) 74(4):682–8. 10.1016/j.jadohealth.2023.08.013 37791924 PMC10960661

[96] Leung AKC Lam JM Hon KL. Premature thelarche: an updated review. Curr Pediatr Rev. (2024) 20(4):500–9. 10.2174/1573396320666230726110658 37496240

[97] Bloomfield V Iseyemi A Kives S. Clinical review: prepubertal bleeding. J Pediatr Adolesc Gynecol. (2023) 36(5):435–41. 10.1016/j.jpag.2023.06.002 37301426

[98] Elhakeem A Frysz M Goncalves Soares A Bell JA Cole TJ Heron J. Evaluation and comparison of nine growth and development-based measures of pubertal timing. Commun Med. (2024) 4(1):159. 10.1038/s43856-024-00580-1 39112679 PMC11306255

[99] Yücel Ekici N Demet Akbaş E Kadir Arslan A. Voice aspects in children with precocious puberty. Int J Pediatr Otorhinolaryngol. (2024) 180:111962. 10.1016/j.ijporl.2024.111962 38657429

[100] Erol U Yücel L Genç H Bolat A Aşık MB. Can multi-dimensional voice program (MDVP) be used as A diagnostic tool for precocious puberty? J Voice. (2024) 38(4):965.e25–e31. 10.1016/j.jvoice.2021.11.001 34857449

[101] Kärkinen J Sorakunnas E Miettinen PJ Raivio T Hero M. The aetiology of extreme tall stature in a screened Finnish paediatric population. eClinicalMed. (2021) 42:101208. 10.1016/j.eclinm.2021.101208 PMC8608868 34849478

[102] Song Y Kong Y Xie X Wang Y Wang N. Association between precocious puberty and obesity risk in children: a systematic review and meta-analysis. Front Pediatr. (2023) 11:1226933. 10.3389/fped.2023.1226933 37635793 PMC10456873

[103] Liu G Guo J Zhang X Lu Y Miao J Xue H. Obesity is a risk factor for central precocious puberty: a case-control study. BMC Pediatr. (2021) 21(1):509. 10.1186/s12887-021-02936-1 34784914 PMC8594221

[104] Tzounakou A-M Stathori G Paltoglou G Valsamakis G Mastorakos G Vlahos NF. Childhood obesity, hypothalamic inflammation, and the onset of puberty: a narrative review. Nutrients. (2024) 16(11):1720. 10.3390/nu16111720 38892653 PMC11175006

[105] Zheng Y Liang J Zeng D Tan W Yang L Lu S. Association of body composition with pubertal timing in children and adolescents from Guangzhou, China. Front Public Health. (2022) 10:943886. 10.3389/fpubh.2022.943886 PMC9428289 36062089

[106] Fan HY Tsai WL Chien KL Hsu SY Huang L Hou JW. Associations of central precocious puberty with blood pressure trajectories: prospective cohort study. Pediatr Res. (2024) 95(4):1147–52. 10.1038/s41390-023-02908-4 38001237

[107] Fan HY Chien KL Huang YT Hsu JBK Chen YY Lai EY. Hypertension as a novel link for shared heritability in age at menarche and cardiometabolic traits. J Clin Endocrinol Metab. (2023) 108(9):2389–99. 10.1210/clinem/dgad104 36810613

[108] Shope C Ritter A Matlock K Wine Lee L. Pathologic acne in pre-pubertal children: a case series and review on when to refer to pediatric endocrinology. Pediatr Dermatol. (2023) 40(1):5–10. 10.1111/pde.15166 36394116

[109] Çolaklar A Fitoz ÖS. Pituitary gland volumes in children with normal endocrine function. Pediatr Radiol. (2023) 53(3):450–60. 10.1007/s00247-022-05505-5 36138218

[110] Pape J Rosolowski M Zimmermann P Pfäffle R Hirsch FW Gräfe D. Acceleration of skeletal maturation in central Europe over the last two decades: insights from two cohorts of healthy children. Pediatr Radiol. (2024) 54(10):1686–91. 10.1007/s00247-024-05994-6 39030392 PMC11377632

[111] Bıyıklı E Helvacıoğlu D Buğdaycı O Tosun BG Turan S Güran T. Evaluating breast ultrasonography as a complementary diagnostic method in girls with central precocious puberty. Pediatr Radiol. (2024) 54(7):1156–67. 10.1007/s00247-024-05934-4 PMC11182864 38717607

[112] Smets AM Sofia C Bruno C Ključevšek D Lobo ML Napolitano M. Abdominal imaging in precocious puberty in girls: can imaging determine onset of puberty? Pediatr Radiol. (2025) 55(4):660–73. 10.1007/s00247-024-05992-8 39069591 PMC11982095

[113] Birbas E Kanavos T Gkrozou F Skentou C Daniilidis A Vatopoulou A. Ovarian masses in children and adolescents: a review of the literature with emphasis on the diagnostic approach. Children (Basel. (2023) 10(7):1114. 10.3390/children10071114 37508611 PMC10377960

[114] Cui HJ Liu P Liu XG Du WZ Wang X. The clinical value of serum-related indexes in differentiating simple premature thelarche from idiopathic central precocious puberty. Pak J Med Sci. (2023) 40(3):467–72. 10.12669/pjms.40.3.7447 PMC10862426 38356806

[115] Jiang M Gao Y Qu T Ji Y Niu Y Zhang J. Dose inhibin B or anti-müllerian hormone relate to precocious puberty in girls? Result of a systematic review and meta-analysis. J Ovarian Res. (2023) 16(1):227. 10.1186/s13048-023-01302-2 37996919 PMC10668349

[116] Hu Y Li J Yuan T Yu T Chen Y Kong H. Exposure to antibiotics and precocious puberty in children: a school-based cross-sectional study in China. Environ Res. (2022) 212(Pt C):113365. 10.1016/j.envres.2022.113365 35490828

[117] Bakhtiani P Srivastava R Geffner ME. Puberty today, gone tomorrow: transient refractory central precocious puberty in a toddler with End-stage kidney disease. Horm Res Paediatr. (2024) 98(1):1–7. 10.1159/000536323 38262375

[118] Mastacouris N Midgette B Strunk A Garg A. Precocious puberty among children and adolescents with hidradenitis suppurativa. JAMA Dermatol. (2024) 160(11):1245–6. 10.1001/jamadermatol.2024.3104 39292442 PMC11411440

[119] Patyra K Löf C Jaeschke H Undeutsch H Zheng HS Tyystjärvi S. Congenital hypothyroidism and hyperthyroidism alters adrenal gene expression, development, and function. Thyroid. (2022) 32(4):459–71. 10.1089/thy.2021.0535 35044245 PMC9048185

[120] Razzaq M Clément F Yvinec R. An overview of deep learning applications in precocious puberty and thyroid dysfunction. Front Endocrinol. (2022) 13:959546. 10.3389/fendo.2022.959546 PMC9632447 36339395

[121] Augsburger P Liimatta J Flück CE. Update on adrenarche-still a mystery. J Clin Endocrinol Metab. (2024) 109(6):1403–22. 10.1210/clinem/dgae008 38181424

[122] Flokas ME Wakim P Kollender S Sinaii N Merke DP. Gonadotropin-Releasing hormone agonist therapy and longitudinal bone mineral density in congenital adrenal hyperplasia. J Clin Endocrinol Metab. (2024) 109(2):498–504. 10.1210/clinem/dgad514 37643900 PMC10795903

[123] Sandru F Petca RC Carsote M Petca A Dumitrascu MC Ghemigian A. Adrenocortical carcinoma: pediatric aspects (review). Exp Ther Med. (2022) 23(4):287. 10.3892/etm.2022.11216 35317446 PMC8908472

[124] Liu P Li ZH Song HC Gong CX Zhang WP. Central precocious puberty should be taken seriously in children with leydig cell tumors of the testis after surgical treatment: a tertiary center experience. Asian J Androl. (2024) 26(6):617–21. 10.4103/aja202445 39119665 PMC11614166

[125] Gaikwad PM Goswami S Sengupta N Baidya A Das N. Transformation of peripheral sexual precocity to central sexual precocity following treatment of Granulosa cell tumor of the ovary. Cureus. (2022) 14(2):e22676. 10.7759/cureus.22676 35371651 PMC8966063

[126] Heo S Shim YS Lee HS Hwang JS. Clinical course of peripheral precocious puberty in girls due to autonomous ovarian cysts. Clin Endocrinol. (2024) 100(1):29–35. 10.1111/cen.14943 37386805

[127] Tripathi M Sheehan JP Niranjan A Ren L Pikis S Lunsford LD. Gamma knife radiosurgery for hypothalamic hamartoma: a multi-institutional retrospective study on safety, efficacy, and complication profile. Neurosurgery. (2025) 96(2):426–37. 10.1227/neu.0000000000003110 38990006

[128] Yuan S Lin Y Zhao Y Du M Dong S Chen Y. Pineal cysts may promote pubertal development in girls with central precocious puberty: a single-center study from China. Front Endocrinol. (2024) 15:1323947. 10.3389/fendo.2024.1323947 PMC10885350 38405141

[129] Zhang L Li X Li C Wang Z Zheng L Qin G. Analysis of the clinical characteristics and pituitary function of patients in central China with rathke’s cleft cysts. Front Endocrinol. (2022) 13:800135. 10.3389/fendo.2022.800135 PMC8919671 35295993

[130] Zhu R Wang L Zhao L Liu X. Central precocious puberty secondary to postoperative craniopharyngioma: two case reports and a literature review. Childs Nerv Syst. (2024) 40(7):2245–50. 10.1007/s00381-024-06406-8 38635073

[131] Zhang D Gu M. Metabolic/endocrine disorders in survivors of childhood-onset and cranial radiotherapy- treated ALL/NHL: a meta-analysis. Reprod Biol Endocrinol. (2023) 21(1):91. 10.1186/s12958-023-01137-y 37794442 PMC10548660

[132] Cheng G Buyken AE Shi L Karaolis-Danckert N Kroke A Wudy SA. Beyond overweight: nutrition as an important lifestyle factor influencing timing of puberty. Nutr Rev. (2012) 70(3):133–52. 10.1111/j.1753-4887.2011.00461.x 22364156

